# Hydrogen is neuroprotective against surgically induced brain injury

**DOI:** 10.1186/2045-9912-1-7

**Published:** 2011-05-18

**Authors:** Jan M Eckermann, Wanqiu Chen, Vikram Jadhav, Frank PK Hsu, Austin RT Colohan, Jiping Tang, John H Zhang

**Affiliations:** 1 Department of Neurosurgery, Loma Linda University Medical Center, 11234 Anderson Street, Loma Linda, CA 92354, USA; 2 Department of Physiology and Pharmacology, Loma Linda University Medical Center, 11234 Anderson Street, Loma Linda, CA 92354, USA; 3 Department of Anesthesiology, Loma Linda University Medical Center, 11234 Anderson Street, Loma Linda, CA 92354, USA

## Abstract

**Background:**

Neurosurgical operations cause unavoidable damage to healthy brain tissues. Direct surgical injury as well as surgically induced oxidative stress contributes to the subsequent formation of brain edema. Therefore, we tested the neuroprotective effects of hydrogen (H_2_) in an established surgical brain injury (SBI) model in rats.

**Materials and methods:**

Adult male Sprague - Dawley rats (weight 300-350g) were divided into three groups to serve as sham operated, SBI without treatment, and SBI treated with H_2 _(2.9%). Brain water content, myeloperoxidase (MPO) assay, lipid peroxidation (LPO), and neurological function were measured at 24 hrs after SBI.

**Results:**

SBI resulted in localized brain edema (p = < 0.001). Hydrogen (2.9%) administered concurrently with surgery significantly decreased the formation of cerebral edema (p = 0.028) and improved neurobehavioral score (p = 0.022). However, hydrogen treatment failed to reduce oxidative stress (LPO assay) or inflammation (MPO assay) in brain tissues.

**Conclusions:**

Hydrogen appears to be promising as an effective, yet inexpensive way to reduce cerebral edema caused by surgical procedures. Hydrogen has the potential to improve clinical outcome, decrease hospital stay, and reduce overall cost to patients and the health care system.

## Introduction

In 2008, close to 800,000 patients underwent neurosurgical procedures in the United States. This figure encompasses emergent, life-preserving surgeries, as well as planned procedures. Neurosurgical operations have the potential to cause unavoidable healthy brain tissue damage as a result through application of pressure, tissue stretching, hemorrhage, and the use of electrocautery [1,2]. This damage occurs despite intraoperative adjunct treatments such as administration of steroids and mannitol, irrespective of the procedure being planned or emergent. Even standard microsurgical technique has the potential to cause damage to the surrounding brain and lead to early complications such as edema and local ischemia [3]. Furthermore so-called "minimally-invasive" operations and endoscopic-assisted procedures are not immune to damage the surrounding structures [4]. Moreover, radiosurgery, which has the least invasive method of addressing intracranial lesions, has the potential to cause severe cerebral edema [5].

Some clinical-used therapeutic agents have shown to be neuroprotective in experimental models [6]. Although pretreatment of the subjects may be an option for planned procedures, it proves to be not useful in the emergent setting for obvious reasons. The ideal therapy would be to start at the time when the neurosurgical intervention is carried out, and is completed at the time when the intervention is completed. Furthermore, this therapy should have a low profile with regard to toxicity, adverse effects, drug-drug interaction, and should also be cost-effective.

Recent data suggests that hydrogen (H_2_) has neuroprotective effects in an experimental model of cerebral ischemia [7]. Inhaled hydrogen was shown to decrease the infarct size by reducing reactive oxygen species formation. We hypothesize that hydrogen, inhaled concomitantly with the neurosurgical intervention, has potential to reduce brain edema and improve neurological function in an established surgical brain injury model [8].

## Materials and methods

This protocol was evaluated and approved by the Institutional Animal care and Use Committee at Loma Linda University, Loma Linda, CA.

### The SBI model

The SBI model in rodents has been described previously [8]. Briefly, the rationale is that a controlled insult - a partial right frontal lobectomy - mimics standard surgical procedures. By using fixed coordinates (see below), the repeatability of this procedure is insured. By creating a lesion close to the right primary sensory and motor cortices, it is possible to observe impairments in neurological function. Both sensory as well as motor systems are affected and are quantifiable with established, objective tests. Brain water content measurement after sacrificing the animal assures an objective, quantitative method to evaluate edema formation.

### Surgical Procedure

Adult male Sprague - Dawley rats (weight 300-350 g) were used for this study. The rodent model of SBI was used as described previously [8]. All animals were intubated and subject to general anesthesia, using 3% halothane for induction and 1% for maintenance. A square cranial window was drilled such that the left lower corner of the square was at the bregma. The dura was opened and reflected to expose the underlying right frontal lobe. Using a sharp blade, two incisions were made along the saggital and coronal planes leading away from the bregma to limit the partial lobectomy to 2 mm lateral to the saggital plane and 1mm rostral to the coronal plane. The depths of the resection extended to the base of the anterior fossa. Hemostasis was achieved by using saline irrigation, packing, and electrocautery. Sham surgery included only craniotomy and replacement of the bone flap without dural penetration. Animals were sacrificed at 24 hours. The 24 hour time point appears to be the time of peak edema formation in rodents, making it thus the prime target for any therapy aimed at edema reduction.

All animals were intubated for sixty minutes regardless of the length of the operation and group. This allows for a constant in the length of intubation and hydrogen administration. No surgery exceeded sixty minutes.

### Treatment Groups

H_2 _was administered via the endotrachial tube. A tank premixed with 2.9% hydrogen, 21% oxygen and nitrogen balance was used for the treatment group and sham (2.9%, Praxair, CA, USA). The control group received 21% oxygen and nitrogen balance. Treatment time consisted of 60 minutes for all groups. The tubing was checked for leaks and adequate concentrations of hydrogen at the endotrachial tube prior to the procedure using a Hydrogen-meter (H_2 _Scan, CA, USA).

### Brain Water Content

The animals were sacrificed under deep anesthesia at 24 hours after frontal lobe injury. The brains were removed and divided into frontal ipsilateral, frontal contralateral, parietal ipsilateral, parietal contralateral, brain stem and cerebellum on ice. These parts were weighed immediately (wet weight) and weighed again after drying in an oven at 105ºC for 48 hours (dry weight) as described previously [8]. The percent of water content was calculated as [(wet weight - dry weight)/wet weight] × 100%. The number of animals used in each group for brain edema study was control (n = 12), sham (n = 8), hydrogen-treated (n = 12).

### Myeloperoxidase Assay

Methodology for the myeloperoxidase (MPO) assay has been described previously [9]. Briefly, frozen samples (n = 18) were cut into 0.1-0.2 g pieces and homogenized with Tris-HCl and Na^+ ^buffer. The samples were then centrifuged at 14,000 g for 30 minutes. The supernatants were discarded and the samples were resuspended in 0.5%HTAB/K^+ ^buffer. Several cycles of freezing in liquid nitrogen followed by sonication at 25°C were performed, before spectrophotometrical analysis at 460 nm. The value was divided by the exact weight of the sample and expressed in MPO activity Unit/gram.

### Lipid Peroxidation Assay

The method for Lipid Peroxidase (LPO) Assay has been described in detail previously [10]. Briefly, animals (n = 18) were anesthetized and brain samples were collected at 24 hours after SBI. The level of lipid peroxidation products (malondialdehyde [MDA]) in the right cerebral cortexes was measured using a LPO-586 kit (OxisResearch; Portland, OR). Right frontal cerebral cortexes were homogenized in 20 mmol/L phosphate buffer (pH 7.4) with 0.5 M butylated hydroxytoluene in acetonitrile. The homogenates were centrifuged at 20,800 g for 10 min at 4°C and the supernatants were collected. Protein concentration was measured by DC protein assay (Bio-Rad) and the samples were reacted with a chromogenic reagent at 45°C for 60 min. After incubation, the samples were centrifuged at 20,800 g for 10 min at 4°C and supernatants were measured at 586 nm. The level of MDA was calculated as picomoles per milligram protein according to the standard curve.

### Neurological Scoring

We used a 21-point score adapted from the one developed for stroke by Garcia et al. [11] as presented in Table 1. Furthermore, we used the wire-hang and beam balance tests for assessment of balance, strength, and coordination. The wire-hang and beam balance test were assessed based on a three point scale with dependence on time and/or reaching the platform. 0 = less than 15 seconds, no movement towards platform, 1 = less than 40 seconds, no movement towards platform, 2 = less than or equal to half distance to platform within 40 seconds, and 3 = reaching platform within 40 seconds. Three trials were performed while animals were receiving a five minute rest period in between trials. The final score was the average of three trials.

**Table 1:** The Garcia Neurological Scoring Scale

	Test	3	2	1	0
**I**	Spontaneous activity	3-4 walls, 1-2 walls plus raise on hindlimbs	1-2 walls	Minimal movement	Akinesia

**II**	Side stroking	Bilateral brisk	Bilateral weak or ipsilateral strong and contralateral weak	Unilateral	No response

**III**	Vibrissae touch	Bilateral brisk	Bilateral weak or ipsilateral strong and contralateral weak	Unilateral	No response

**IV**	Limb symmetry	Forelimb and hindlimb extended	Mid flexion of forelimb	Contralateral forelimb flexed with hindlimb extended	Contralaterally flexed

**V**	Lateral turning	Bilateral turning >450	Bilateral turning <450	Unilateral turning	No turning

**VI**	Forelimb walking	Brisk forward	Moves toward on side	Moves in circle	Cannot move forelimbs

**VII**	Climbing	Climbs to top	Climbs up weak or strong but not to top	Moves laterally or downward	Weak grip and falls off

Note: The neurological evaluation system adapted from Garcia et al. This system was applied to all groups in a blinded fashion.

### Statistical Analysis

All data were collected in SigmaPlot and analyzed with SigmaStat (both Systat Software, Inc, San Jose, CA). The one-way ANOVA test was used to calculate statistical significance between samples. P value below 0.05 was considered as statistically significant.

## Results

### Outcome after SBI

**Figure 1:**
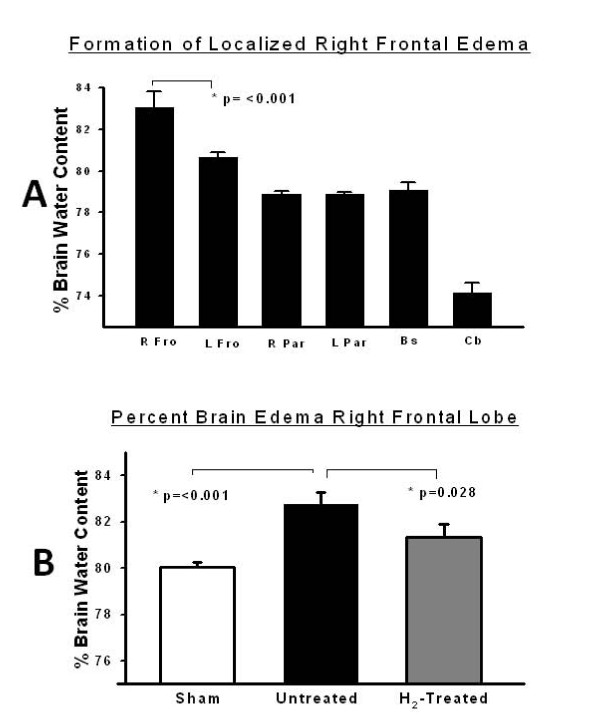
**Hydrogen on brain water content**. (A) Localized edema formation localized in the right frontal lobe demonstrating the efficacy of the SBI model. The right frontal lobe exhibit the highest water content in relation to all other brain sections (n = 12). The bars represent the standard error. (B) Percent brain edema in the right frontal region comparing the untreated partial right frontal lobectomy group to the hydrogen-treated partial right frontal lobectomy group (n = 32). The bars represent the standard error.

SBI results in localized brain edema (Figure 1A). This simply demonstrates the validity of the model. Hydrogen (2.9%) administered concurrently with surgery significantly decreased the formation of cerebral edema (Figure 1B) evidenced by the decrease in brain water content.

**Figure 2:**
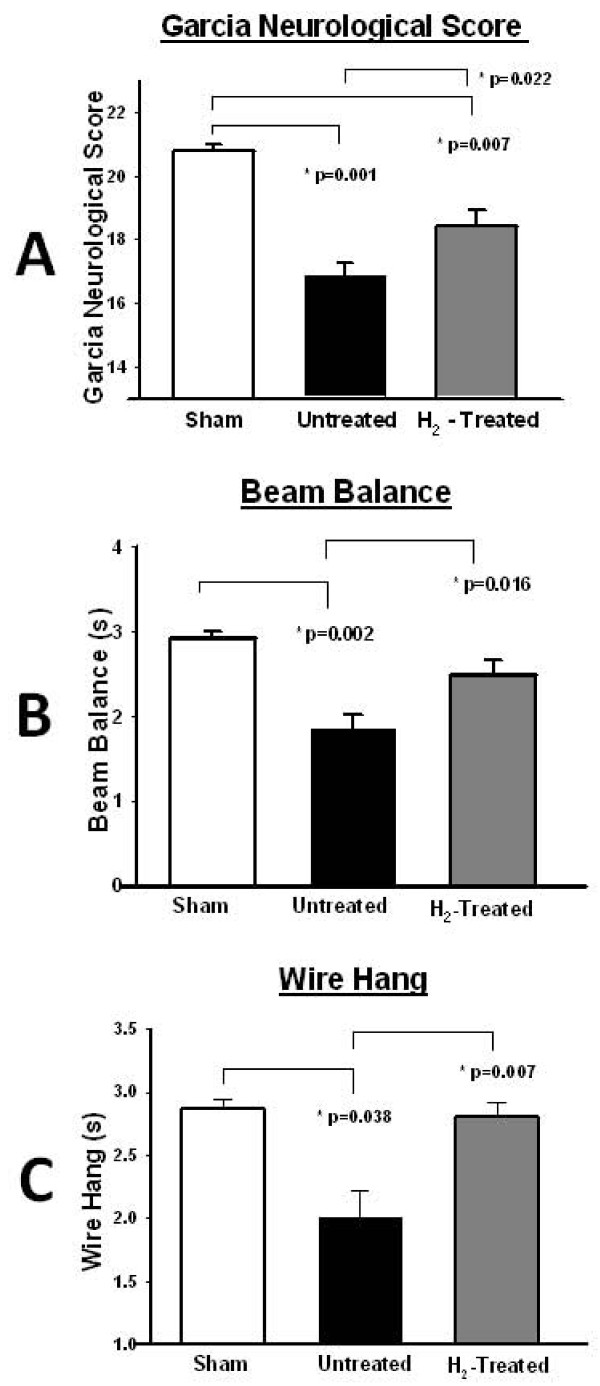
**Hydrogen on neurological function**. (A) Neurological outcome scores adapted from Garcia et al. showing the difference between hydrogen-treated animals and untreated as well as sham animals (n = 34). The bars represent the standard error. (B) The beam balance test, quantifying balance and coordination, showing the difference in hydrogen-treated animals versus controls (n = 34). The bars represent the standard error. (C) The wire hang test, quantifying coordination and strength, demonstrating the difference between hydrogen-treated animals and controls (n = 34). The bars represent the standard error.

Twenty-four hours after the operation, all animals underwent standardized, blinded neurological evaluation. The sensory as well as motor evaluation showed a significant improvement in the hydrogen treatment groups compared to the control (Figures 2A-C).

### Chemical Analysis

**Figure 3:**
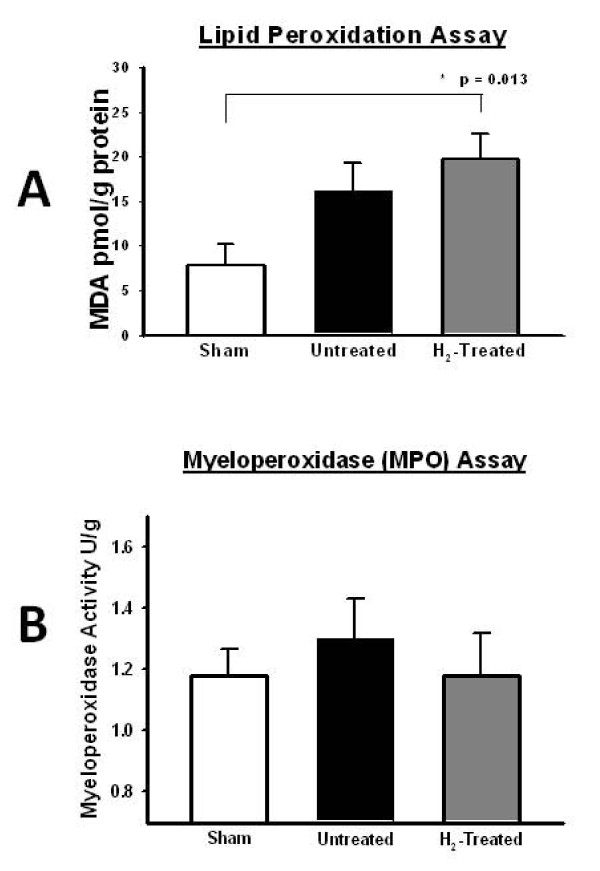
**Hydrogen on brain lipid peroxidation and MPO**. (A) The Lipid peroxidation (LPO) assay showing the increased oxidation in the Hydrogen-treated group compared to sham (n = 18). The bars represent the standard error. (B) The myeloperoxidase (MPO) assay showing no difference in inflammation in all study groups (n = 18). The bars represent the standard error.

Lipid peroxidation (LPO) assay did not show a decrease in oxidative stress in the hydrogen group. Paradoxically, the amount of oxidative stress increased compared to the sham and control group (Figure 3A).

Myeloperoxidase (MPO) assay was unable to demonstrate a significant attenuation of the inflammation associated with SBI. There was no statistically significant difference between treatment and control groups (Figure 3B).

## Discussion

Vasogenic edema, resulting after breakdown of the blood brain barrier, is often associated with mass lesions and is encountered frequently clinically [12]. This happens not only during pathological processes, such as a tumor growth [13], but also occurs in normal healthy brain tissues caused by a neurosurgical manipulation [2]. We have observed in this study that localized brain edema occurred after SBI in rats, which leads to poor neurological function, accompanied by increase of lipid peroxidation in the injured brain tissues.

Inhaled 2.9% hydrogen during 60 minutes of surgical procedures significantly reduced brain edema and improved neurological function. This study provides evidence that inhaled hydrogen could be an easy, economic, convenient, and applicable approach for anesthesiologists and neurosurgeons to reduce surgery induced brain edema and tissue injury.

Even though we have observed positive results on the clinical outcomes, we could not confirm the mechanisms of hydrogen induced neuroprotection reported by others [7]. It has been postulated that hydrogen reacts with reactive oxygen species causing a decreases in oxidative stress at the tissue level and thus decreasing the amount of blood brain barrier breakdown [7]. The decrease in blood brain barrier breakdown in turn results in the decreases vasogenic edema formation [14]. We measured lipid peroxidation, the final outcome of oxidative stress [15]. SBI produced localized brain edema accompanied by an increase of lipid peroxidation in this rat model. Inhaled hydrogen reduced formation of brain edema, but not only did it fail to reduce, but rather it enhanced lipid peroxidation significantly. This observation is not consistent with a previous report that hydrogen is a scavenger of free radicals [7]. Somehow, our results in this study are consistent with one of our previous publications that hydrogen failed to reduce lipid peroxidation in a neonatal hypoxia-ischemia rat pup model [16]. Even though it is possible that hydrogen scavenges selectively hydroxyl radicals [7], the overall effect of hydrogen on lipid peroxidation is either ineffective [16] or potentiating, as observed in this study. It is not clear whether there is an interaction between halothane and hydrogen. However, Ohsawa et al. demonstrated H_2 _protects cultured cells against oxidative stress, without the existence of halothane [7]. It suggests that hydrogen-induced neuroprotection is halothane-independent.

Because hydrogen failed to reduce lipid peroxidation, we examined its effect on inflammation. It has been reported that inflammation contributes to secondary brain injury in stroke and traumatic brain injury [17], and anti-inflammatory strategy achieved marked brain protection in different experimental models [18]. Therefore it is highly likely that surgical brain injury is partially mediated by inflammatory responses in rats and in mice. Similar observations of surgical induced brain injury mediated by inflammation have been reported. Because neural inflammation can be detected by MPO assay [9], we measured MPO in sham, SBI, and SBI treated with hydrogen rats. We found, however, that MPO was not increased after surgical brain injury and inhaled hydrogen did not increase or decrease MPO activity.

Inhaled hydrogen gas has been proven to be safe in humans (divers) at low concentrations [19]. Even though pure hydrogen is safe, the threshold for combustion of hydrogen if mixed with oxygen lies at four percent [20]. It has been shown that with 2-4% of H_2 _inhalation significantly reduced infarct volume against middle cerebral artery occlusion [7]. In our study with 2.9% and for only one hour, we are clearly observing a phenomenon of decreasing cerebral edema formation as well as improved functional outcome in animals treated with inhaled hydrogen. Therefore, hydrogen appears to be promising as an effective, yet inexpensive way to reduce cerebral edema. The non-toxicity of low-dose hydrogen [3] coupled with its availability and ease of administration makes a clinical translation very simple and foreseeable in the near future. Based on the results observed in this study, perioperative use of hydrogen has the potential to improve clinical outcome, decrease overall healthcare cost and length of hospital stay, as well as increased patient satisfaction. Further investigations are necessary to elucidate the exact mechanism in which hydrogen exerts its protection on neuronal tissues. Our study only assessed the effects of H_2 _treatment acutely following surgical-induced brain injury. Since neurosurgical operations have been reported to cause postoperative cognitive dysfunction [21], it will be interesting to investigate the long term effects of H_2 _inhalation in the future, such as cognitive and memory functions following SBI.

## Competing interests

The authors declare that they have no competing interests.

## Authors' contributions

JE carried out all the animal surgery, sample collection and drafted the manuscript. WC carried out all the neurological scoring tests and lipid peroxidation assay. VJ participated in the design of the study. FH participated in the study design and coordination. AC participated in the design of the study and coordination. JT participated in the study design. JZ participated in the design of the study and manuscript drafting. All authors read and approved the final manuscript.
